# Retraction Note: MiR-140 targets RAP2A to enable the proliferation of insulin-treated ovarian granulosa cells

**DOI:** 10.1186/s13048-023-01118-0

**Published:** 2023-02-16

**Authors:** Zhengfang Xiong, Bing Li, Wenjuan Wang, Xianghui Zeng, Binye Li, Shengyan Jian, Liyun Wang

**Affiliations:** 1 Reproductive Medical Center, Qinghai Provincial People’s Hospital, No. 2, Gonghe Road, Xining, 810007 Qinghai China; 2 Department of General Surgery, Qinghai Provincial People’s Hospital, Xining, 810007 Qinghai China

**Retraction Note: J Ovarian Res 13, 13 (2020)**

**https://doi.org/10.1186/s13048-020-0611-4**

The Editors-in-Chief have retracted this article. After publication, concerns were raised regarding similarities between the western blot (Figs. 5c and 6b) and flow cytometry (Figs. 3a and 6e) data presented in this article and a number of other articles by different authors that were under consideration in different journals within a similar time frame [1–5]. Further checks by the Publisher identified inconsistencies in flow cytometry gating in Figs. 3a and 6e, as well as in the data presented in Table S1.

The Editors-in-Chief therefore no longer have confidence in the presented data.

Zhengfang Xiong, Bing Li, Wenjuan Wang, Xianghui Zeng, Binye Li and Liyun Wang agree to this retraction. Shengyan Jian has not responded to any correspondence from the editor or publisher about this retraction.
